# Prevalence of chronic kidney disease and associated factors among patients with underlying chronic disease at Dessie Referral Hospital, East Amhara Region, Ethiopia

**DOI:** 10.3389/fepid.2023.1154522

**Published:** 2023-07-18

**Authors:** Ahmed Ali, Kebadnew Mulatu, Sefineh Fenta Feleke, Gizachew Tadesse Wassie

**Affiliations:** ^1^ Department of Epidemiology and Biostatistics, School of Public Health, College of Medicine and Health Sciences, Bahir Dar University, Bahir Dar, Ethiopia; ^2^ Department of Public Health, College of Health Sciences, Woldia University, Woldia, Ethiopia

**Keywords:** Chronic kidney disease, glomerular filtration rate, proteinuria, hypertension and diabetes mellitus, urology

## Abstract

**Background:**

Chronic kidney disease is defined as a reduction in glomerular filtration rate below 60 ml/min per 1.73 m^2^ and presence of albuminuria over a period of time. Globally, 10%–15% of populations are affected by chronic kidney disease. Studies conducted in Jimma, Addis Ababa, and the Tigray region were conducted on a single chronic disease and did not include human immune viruses. In addition, there has been no such study conducted in the Amhara region. Therefore, the aim of this study was to determine the magnitude and associated factors of chronic kidney disease among chronic patients who are followed up at an outpatient department.

**Methods:**

An institutional-based cross-sectional study of 480 chronic patients was conducted at Dessie Referral Hospital, Dessie, Ethiopia between 15 March and 16 April 2020. Data were entered into Epidata and exported to SPSS version 25 for analysis. Binary logistic regression models were performed to identify factors associated with chronic kidney disease. The variables with a *p*-value ≤0.25 were considered to be a candidate for multivariable logistic regression. A *p*-value ≤0.05 was considered a statistically significant association.

**Results:**

The magnitude of chronic kidney disease among the study participants was 21.3%. Variables such as hypertension [adjusted odds ratio (AOR): 2.6, 95% CI: 1.58–4.27], use of non-steroidal anti-inflammatory drugs (AOR: 2.4, 95% CI: 1.41–3.97), smoking (AOR: 4.4, 95% CI: 2.65–7.34), routine physical activity (AOR: 0.6, 95% CI: 0.35–0.94), and obesity (AOR: 3.0, 95% CI: 1.76–5.05) were significantly associated with the chronic kidney disease.

**Conclusion:**

This study found that the magnitude of chronic kidney disease in the study area was high. Hypertension, use of non-steroidal anti-inflammatory drugs, smoking, routine physical activity, and obesity were found to be significant factors for chronic kidney disease.

## Introduction

Chronic kidney disease (CKD) is defined as a reduction in glomerular filtration rate (GFR) below 60 ml/min per 1.73 m^2^ and the presence of albuminuria over a period of time or an indication of abnormalities in the kidney structure or function in those who had known CKD (1). In patients with CKD, excess fluid and waste materials from the blood remain in the body and are not excreted from the body, leading to severe health problems (2). Chronic kidney disease is a silent killer. A person can lose up to 90% of kidney function before experiencing any signs and symptoms. Most people have no symptoms until CKD is advanced. Untreated kidney failure is life-threatening, so the early detection of falling kidney function is crucial because it allows for suitable treatment before the kidney is damaged (3, 4).

Chronic kidney disease is an important and common public health problem in NCDs. It affects as much as 10%–15% of the world's population (5). Globally, CKD represents a major public health issue that can consume substantial financial and social resources (6, 7). Currently, more than 2 million people globally receive treatment with dialysis or kidney transplants to stay alive; of them, 20% are treated in 100 low-income countries, which make up half of the world’s population (8).

In 2017, the global burden of disease (GBD) reported that CKD ranks the 16th leading cause of premature death and is estimated to be the fifth leading cause of premature death by 2040 (9). The pooled prevalence of CKD in Africa and sub-Saharan Africa was 10% and 14%, respectively (10). Kidney disease is associated with a great economic burden. High-income countries typically spend more than 2%–3% of their annual healthcare budget on the treatment of end-stage renal disease (ESRD) (11).

Next to cardiovascular complications, CKD is a serious public health issue among people with type 2 diabetes (T2DM). Patients with type 2 diabetes are more likely to require recurrent hospital stays and are at greater risk of increased mortality (12). CKD is a well-recognized and serious complication of diabetes, and diabetes is one of the most common causes of CKD, with up to 44% of patients with CKD affected by diabetes (13). The prevalence of CKD among patients with hypertension (HTN) was 21.1% in Tigray (14), 46.9% in Gahanna (15), and 17.6% in Northwest Ethiopia referral hospitals (16). Geographically, the prevalence of CKD linked to human immunodeficiency virus (HIV) varies, ranging between 2% and 38% due to genetic variation, the start of anti-retroviral therapy (ART), and the various definitions of CKD in each region (17). The global prevalence of chronic kidney disease among persons living with HIV (PLHIV) is 6.4%. This prevalence varies by continent: 7.9% in Africa, 7.1% in North America, 5.7% in Asia, and 3.7% in Europe (18).

CKD is associated with an impaired quality of life and substantially reduces life expectancy at all ages. It is also associated with increased premature death from cardiovascular disease and progressive loss of kidney function that can lead to kidney failure or ESRD. Data on the prevalence of CKD are limited; however, a few studies suggest that renal illness has emerged as a serious public health issue in Ethiopia. A cross-sectional study estimates that 12.2% of Ethiopians have CKD, and that the number has increased recently along with instances of diabetes and high blood pressure. Up to 41% of people under the age of 35 years and 62% of men have CKD in Ethiopia (19).

In low- and middle-income countries, most people with kidney failure have insufficient access to life-saving dialysis and kidney transplantation. The cost of treating CKD and its complications is unaffordable to the government and individuals in many part of the world, including Ethiopia (6, 20). It is of great importance for policymakers to know the magnitude of CKD in order to prevent it, as it is related to high morbidity and mortality and the high cost of renal replacement therapy. The patients in this study would be expected to benefit from the findings of this research at large because they receive treatment for CKD, and counseling and health education on the risk factors of CKD. It serves as baseline data for further studies in this area.

There are studies conducted in Jimma, Tigray, and Addis Ababa, but those studies were conducted on a single chronic disease and did not include human immune viruses. In addition, there was no such study conducted in the Amhara region. Therefore, the aim of this study was to assess the magnitude and associated factors of chronic kidney disease among chronic patients (HTN, diabetes mellitus (DM), HIV/AIDS, two of these conditions concurrently, or all of the above) who are followed up at an outpatient department.

## Methods

### Study setting

The present study was conducted at Dessie Referral Hospital in Dessie town. Dessie town is located in the Amhara region of northeastern Ethiopia between 11°8′N and 39°38′E latitude and 11.133°N 20′ and 39.633°E longitude, with an elevation between 2,470 m and 2,550 m above sea level. Dessie town is located 401 km away from Addis Ababa and 480 km from Bahirdar. Dessie Referral Hospital provides different services to both inpatients and outpatients, such as curative and palliative services.

### Design and study period

A facility-based cross-sectional study was conducted between 15 March and 16 April 2020 at the hypertension, ART, and diabetes outpatient clinics at Dessie Referral Hospital.

### Study population

The study population included all adults aged ≥18 years with chronic disease who had regular follow-ups at the Dessie Referral Hospital outpatient clinic during the study period between 15 March and 16 April 2020.

### Eligibility criteria

#### Inclusion criteria

Adult patients (aged ≥18 years) with chronic disease who had regular follow-ups at the outpatient clinic were included in the study. Those who gave informed consent and agreed to provide samples of urine and blood were enrolled.

#### Exclusion criteria

Pregnant women and individuals who had an acute febrile illness with the possibility of interfering with results were excluded.

### Sample size determination

The sample size was calculated based on a single population proportion formula using the following assumptions: 95% confidence interval; 4% margin of error; and the value of proportion was taken from a previous study done in Jimma zone on the prevalence and risk factors of CKD among adult hypertensive patients, which showed 26% of CKD (13) and 10% of non-respondent rates. The total sample size consisted of 509 individuals and a systematic random sampling technique was applied to select study participants.

### Sampling procedure

The study was conducted at Dessie Referral Hospital, one of the largest hospitals in Dessie town providing different services to patients. Dessie Referral Hospital was selected using a convenient method. The study participants were selected using a systematic random sampling technique. The total number of chronic patients who had regular follow-ups at the HTN, DM, and ART outpatient departments was 1,075. Then *k* was calculated by dividing 1,075 by the sample size, which was 2. The data were collected from the study participants via face-to-face interviews, a questionnaire, and secondary data from the patient cards.

### Data collection instruments

Data were collected using a structured interview-administered questionnaire and patient chart review. The questionnaire was initially prepared in English, then translated into the local language (Amharic), and translated back into English. One week before the actual data collection period, the questionnaire was pre-tested on 5% of the total sample size of patients identified in Boru-Meda Hospital, which has a similar setup to the study area.

### Study variables

#### Dependent variable

The presence or absence of chronic kidney disease was a dependent variable in the study.

#### Independent variables

Socio-demographic factors, history of diabetes, history of hypertension, obesity, smoking habits, alcohol consumption, level of physical activity, use of traditional medicine, and family history of DM and hypertension (HTN) were independent variables.

### Operational definitions

Operational definitions were as follows:

• *Patients with underlying chronic disease:* patients who have HTN, diabetes mellitus (DM), HIV/AIDS, two of these conditions concurrently, or all of the above.
• *Smoking status:* Smoking status was assessed by asking, “On how many of the past 7 days did you smoke a cigarette or cigar, even just one puff?” Respondents who reported 0 days were considered a non-smoker. All others were categorized as smokers (5).
• *Alcohol use:* Alcohol use was determined using the CAGE international screening tool to discuss a patient's alcohol use/abuse. Each response to the four CAGE questions is scored in points: either 0 points for “no” or 1 point for “yes.” If a participant scores ≥2, they are considered to be an alcohol user (21).
• *Non-steroidal anti-inflammatory drug (NSAID) use:* If a person takes diclofenac, ibuprofen, indometcin, or aspirin for at least 2 weeks, they are considered an NSAID user (1).
• *Proteinuria:* Participants were considered to have proteinuria if their laboratory results showed +1 and above for the urine dipstick test (1).

### Measurement tools

#### Estimation of glomerular filtration rate

Estimated GFR is used to assess how well the kidneys are functioning. The test estimates the volume of blood that is filtered by the kidney over a given period of time. The normal value of GFR is 120–130 ml/min.

The eGFR in the Cockcroft Gault formula is calculated as follows:

$$\mathrm{GFR}=\frac{\left(140-\mathrm{age})\times \mathrm{lean}\;\mathrm{body}\;\mathrm{weight}\;(\mathrm{kg}\right)}{\mathrm{Serum}\;\mathrm{creatinine}\;(\mathrm{mg}/\mathrm{dl})}\times 72$$

The value is multiplied by 0.85 in women to account for smaller muscle mass. In this equation, GFR is expressed in ml/min per 1.73/m^2^ and serum creatinine (SCr) is expressed in mg/dl.

#### Anthropometric measurements

Height was measured using a stadiometer and weight was recorded using a digital column scale (Seca 701; Hamburg, Germany) with patients barefoot and wearing light clothes. The body mass index (BMI) was calculated as the weight in kilograms divided by the height in meters squared (kg/m^2^). The patient's height was measured in a standing position. All anthropometric measurements were performed twice and their average was used for analysis.

### Data processing and analysis

Data completeness was checked and entered into Epidata Manager version 4.2 and then exported into SPSS version 23 for data analysis. Once the data were cleaned and coded, descriptive statistics were calculated to describe the data. For descriptive statistics, categorical variables were reported as a proportion, whereas continuous variables were reported as mean ± SD, when distributions were considered approximately normal. A variable with a *p*-value of ≤0.25 in the bivariate analysis was considered to be a candidate for multivariable logistic regression methods. A *p*-value ≤0.05 indicated a statistically significant association between independent variables and chronic kidney disease. The Hosmer–Lemeshow goodness of fit test was used, with a result of 0.221.

### Data quality assurance

Data quality was checked and performed daily according to the laboratory's protocol. Training was given for data collectors and supervisors to enable them to have a common understanding of the objectives of the study and each of the questions in the questionnaire. Data were checked daily for completeness, consistency, accuracy, and clarity. Daily supervision was carried out by the supervisors and the principal investigator.

## Results

### Socio-demographic characteristics

Of the 509 sample sizes, a total of 480 chronic patients were interviewed, with a response rate of 94.3%. The mean age of the study participants was 50 ± 10.78 years (age range: 25–85 years). We intended to include individuals aged ≥18 years, but 25 years was the youngest age we found. A total of 332 (69.2%) respondents were urban dwellers and 301 (62.7%) were women. With regard to marital status, 374 (77.9%) of the study participants were currently married. In total, 290 (60.4%) of the study participants were self-employed. With regard to educational status, 165 (34.4%) of the study participants had no formal schooling and 74 (15.4%) were educated to college level and above (Table 1).

**Table 1:** Descriptive statistics of the socio-demographic characteristics of study participants at Dessie Referral Hospital in Dessie town, Amhara region, May 2020 (*N* = 480).

Variables	Category	*N* (%)
Sex of respondent		
	Male	179 (37.3)
	Female	301 (62.7)
Place of residence		
	Urban	332 (69.2)
	Rural	148 (30.8)
Marital status		
	Currently Married	374 (77.9)
	Separated	45 (9.4)
	Widowed	36 (7.5)
	Divorced	25 (5.2)
Main occupation		
	Government employed	54 (11.3)
	Non-government employed	20 (4.2)
	Self-employed	290 (60.4)
	Student	11 (2.3)
	Homemaker	105 (21.9)
Highest level of education		
	No formal schooling	165 (34.4)
	Primary school completed	157 (32.7)
	Secondary and preparatory school completed	84 (17.5)
	College and above	74 (15.4)

### Medical disease characters

Among the study participants, 170 (35.4%) had DM and 270 (56.3%) were taking their medication. Furthermore, 140 (82.4%) had type 2 DM. Of the 480 study participants, 179 (37.3%) had hypertension. Of the patients, 206 (42.9%) and 66 (36.9%) had a family history of kidney disease and hypertension, respectively. In total, 244 (50.8%) of the study participants were obese. Of the study participants, 246 (51.2%) and 210 (43.8%) had taken traditional medicine and non-steroidal anti-inflammatory drugs for their diseases, respectively (Table 2).

**Table 2:** Clinical characteristics of study participants attending at Dessie Referral Hospital, Amhara region, May 2020.

Variables	Category	*N* (%)
Have you ever been diagnosed for Diabetic Mellitus? (*n* = 480)		
	No	310 (64.6)
	Yes	170 (35.4)
Have you been diagnosed with hypertension? (*n* = 480)		
	No	301 (62.7)
	Yes	179 (37.3)
Family history of hypertension (*n* = 179)		
	No	63 (35.2)
	Yes	66 (36.9)
	Don't known	50 (27.9)
Have you been diagnosed with HIV/AIDs? (*n* = 480)		
	No	321 (66.9)
	Yes	159 (33.1)
Status of obesity (*n* = 480)		
	No	236 (49.2)
	Yes	244 (50.8)
Have you used non-steroidal anti-inflammatory drugs? (*n* = 480)		
	No	270 (56.3)
	Yes	210 (43.8)

### Behavioral factors

According to this study, 118 (24.6%) of the study participants were current cigarette smokers and 73 (61.9%) of them smoked an average of 11–20 cigarettes daily. Of the study participants, 260 (54.2%) were alcohol drinkers and 306 (63.7%) chewed Khat. Regarding physical activity, 300 (62.5%) study participants did routine physical activity. Of those doing routine physical exercise, 190 (63.3%) and 135 (45%) did up to 1 h of exercise per each program on 2–4 days per week (Table 3).

**Table 3:** Behavioral factors for CKD among chronic patients attending at Dessie Referral Hospital in Dessie town, Amhara region, May 2020.

Variables	Category	*N* (%)
Current smoking status (*n* = 480)		
	No	362 (75.4)
	Yes	118 (24.6)
Average number of cigarettes per day (*n* = 118)		
	10 or less	17 (14.4)
	11–20	73 (61.9)
	21–30	28 (23.7)
Alcohol drinking status (*n* = 480)		
	No	220 (45.8)
	Yes	260 (54.2)
Routine physical exercise (*n* = 480)		
	No	180 (37.5)
	Yes	300 (62.5)
Number of physical exercise sessions per week (*n* = 300)		
	<2	93 (31.0)
	2–4	190 (63.3)
	≥5	17 (5.7)
Walking by foot for more than one hour daily (*n* = 480)		
	No	246 (51.2)
	Yes	234 (48.8)

### Magnitude of CKD among chronic patients

The magnitude of CKD was calculated using the Cockcroft–Gault formula, which uses the variables age, weight, sex, and serum creatinine to calculate the eGFR. In all, a total of 102 (21.3%) participants had chronic kidney disease.

### Associated factors for chronic kidney disease

In the bivariable analysis, 10 variables, such as sex of the respondent, place of residence, diagnosis of hypertension, use of non-steroidal anti-inflammatory drugs, cigarette smoking, doing routine physical exercise, number of days eating fruit, walking on foot for at least 1 h daily, eating bedtime snacks three times per week, and obesity, were found to be associated factors of chronic kidney disease (*p*-value ≤0.25). However, in a multivariable analysis, a diagnosis of hypertension, use of non-steroidal anti-inflammatory drugs, cigarette smoking, failing to do routine physical exercise, and obesity remained statistically significant predictors of chronic kidney disease (Table 4).

**Table 4:** Factors associated with chronic kidney disease among underline chronic patients attending at Dessie Referral Hospital, Amhara Region, May 2020.

Variables		Prevalence of CKD		COR(95%CI)	*P*-value	AOR(95%CI)	*P*-value
		Yes (%)	No (%)				
Sex	Male	30 (0.17)	149 (0.83)	1		1	
	Female	72 (0.24)	229 (0.76)	1.62 (0.97–2.51)	0.189	1.4 (0.79–2.31)	0.267
Place of residence	Urban	77 (0.23)	255 (0.77)	1		1	
	Rural	25 (0.15)	123 (0.83)	0.71 (0.38–1.05)	0.230	1.14 (0.55 −2.01)	0.874
Diagnosed with HTN	No	41 (0.14)	260 (0.86)	1		1	
	Yes	61 (0.34)	118 (0.66)	3.30 (2.09–5.15)	0.002	2.43 (1.44–3.92)	0.001
Taking NSAID	No	40 (0.15)	230 (0.85)	1		1	
	Yes	62 (0.30)	148 (0.70)	2.40 (1.54–3.77)	0.032	2.41 (1.42–4.02)	0.001
Smoking status	No	49 (0.13)	313 (0.87)	1		1	
	Yes	53 (0.45)	65 (0.55)	5.21 (3.25 −8.35)	0.027	4.30 (2.56–7.09)	0.000
Routine physical activity	No	53 (0.3)	127 (0.7)	1		1	
	Yes	49 (0.16)	251 (0.84)	0.55 (0.30 −0.73)	0.011	0.64 (0.34–0.92)	0.022
Walking by foot for at least one hour daily	No	57 (0.23)	189 (0.77)	1		1	
	Yes	45 (0.19)	189 (0.81)	0.81 (0.51–1.23)	0.190	0.80 (0.51–1.39)	0.512
Number of days eating fruit	<2	48 (0.22)	166 (0.78)	1		1	
	2–4	47 (0.21)	178 (0.79)	0.92 (0.58 −1.44)	0.156	1.12 (0.62 ­−1.85)	0.795
	>4	7 (0.17)	34 (0.83)	0.70 (0.31 −1.76)	0.241	0.94 (0.33 −2.41)	0.827
Eating bedtime snacking 3 times/week	No	48 (0.24)	155 (0.76)	1		1	
	Yes	54 (0.19)	223 (0.81)	0.82 (0.53 −1.28)	0.132	0.91 (0.5 1– 1.42)	0.543
BMI categories	Normal	18 (0.14)	111 (0.86)	1		1	
	Overweight	12 (0.11)	94 (0.89)	0.81 (0.36–1.72)	0.217	0.82 (0.35–1.90)	0.646
	Obesity	72 (0.29)	173 (0.71)	2.60 (1.45–4.53)	0.023	3.01(1.60–5.76)	0.001

COR, crude odds ratio; AOR, adjusted odds ratio.

## Discussion

The magnitude of CKD in this study was higher than that in the studies conducted in the United Arab Emirates, Pakistan, Thailand, Brazil, India, urban Cameroon, sub-Saharan Africa, western India, Zewuditu Memorial Hospital (Addis Ababa), and the general population of Africa (22–31). This discrepancy was due to the occurrence of several health transitions that can shape the health of the community, such as epidemiologic, rural to urban, and nutritional. The demographic transition promotes a longer life expectancy, which creates ageing populations. The rapid changes in the eating habits of the population towards a high-fat, high-sugar “western” diet brought about by the nutritional transition are exacerbated by the dramatic reductions in non-leisure physical activity brought about by urbanization. These different health transitions combine to have a major impact on the development and progression of chronic kidney disease (25).

Contrary to this, the magnitude of CKD was lower than that in the studies conducted in south-west Ghana and Tanzania (26, 32). This variation might be due to genetic factors, lifestyle factors, socioeconomic status of society, and patient access to healthcare services**.** Furthermore, the magnitude of CKD was in line with studies completed in Tigray, Jimma, Gondar, and Butajira (14, 33–35).

As for the associated variables, this study found a significant association between hypertension and CKD. This finding was consistent with those from other studies in southeast Brazil, Switzerland, Pakistan, southeast Nigeria, Nigeria, and Cameroon (35–39). This similarity might be due to hypertension being a largely attributed risk factor for CKD. HTN develops early during CKD and is associated with adverse outcomes, including a more rapid loss of renal function. Many studies have shown a relationship between blood pressure and the rate of progression of diabetic and non-diabetic kidney disease. This implies that in all patients with CKD, HTN ought to be controlled to levels that are recommended by the national guidelines (125/75). The reduction of blood pressure can be achieved primarily by dietary salt restriction and using pharmacological agents such as angiotensin-converting enzyme inhibitors and angiotensin receptor blockers (37).

This study also reveals that obesity is significantly associated with chronic kidney disease, which is in line with studies conducted in Luxembourg, southeast Nigeria, Cameroon, sub-Saharan Africa, Gondar, Tigray, and Butajira (37, 38, 39, 40). It is possible that obesity decreased high-density lipoprotein levels and increased low-density lipoprotein, which has a significant effect on cardiovascular disease, diabetes, and hypertension, all important contributors towards CKD (41). However, studies conducted in Switzerland and Addis Ababa, Ethiopia showed that obesity was not significantly associated with chronic kidney disease. This discrepancy might be due to a difference in sample size (39, 42).

According to the findings of this study, CKD was more prevalent in patients taking NSAIDS for a prolonged period than in those who did not take NSAIDS; this was in line with the finding of a study conducted in Nigeria (36). This is best explained by the fact that in addition to the clearance of endogenous waste products, excretion of sodium and water, the kidney is responsible for the metabolism and excretion of exogenously administered drugs, making it susceptible to various types of injury. Drug-related factors are the critical first step to the development of nephrotoxicity. Innate drug toxicity is important because the drug or its toxic metabolite may cause kidney injury by impairing renal hemodynamics, direct cellular injury, and osmotic injury. Large doses, extended drug exposure, and nephrotoxic drug combinations further augment nephrotoxicity (37). NSAIDs are widely used to treat pain, fever, and inflammation, which are associated with various clinical kidney syndromes. It has been estimated that 1%–5% of patients who ingest NSAIDs develop some form of nephrotoxicity (1). However, this was not similar to the results of a study carried out in Addis Ababa (36, 39).

The present study reveals that routine physical activity can have a protective effect against chronic kidney disease, which was in agreement with studies conducted in Ghana, Spain, and Japan (25, 40, 43). This implies that being physically active plays a vital role in ensuring health and well-being. In individuals with type 2 diabetes, exercise improves glucose tolerance and insulin sensitivity, which in turn decreases the development of CKD. In addition, routine physical activity has been shown to improve body composition (reduced abdominal adiposity and improved weight control) and lipid profiles (reduced triglyceride levels, increased high-density lipoprotein, and decreased low-density lipoprotein). Physical activity benefits the body by reducing many of the risk factors for CKD and improves the overall quality of life (41). To obtain such benefits, the World Health Organization (WHO) recommends at least 150 min of moderate-intensity and 75 min of vigorous-intensity aerobic physical activity throughout the week for a duration of at least 10 min. However, in this modern era of industrialization and urbanization, numerous environmental factors discourage participation in physical activity, predominantly in terms of transport and occupation (41, 44).

### Limitations and strengths of the study

The present study has some limitations. First, the study participants might have introduced recall bias in remembering their family history of DM, HTN, and kidney disease if the patient had not been living with the family for a long time. Second, eGFR was calculated using a single measure and therefore we might not differentiate acute kidney injury (AKI) from CKD. In addition, as a cross-sectional survey, the associated factors could not be exhausted. The findings should be extrapolated with caution without a representative sample. Finally, the estimation of GFR was performed by using formulae rather than direct measurements. Even though there were limitations, the quality of the data was strictly controlled during data collection and data entry.

## Conclusions

This study revealed a high magnitude of CKD in the study area. Hypertension, cigarette smoking, obesity, and the prolonged use of NSAIDs were the most important shared risk factors and were responsible for the high magnitude of CKD in the study population. Thus, early identification and management of CKD by using eGFR among chronic patients is essential to avoid extra morbidity and mortality. Policies that guide the screening of patients with chronic and high-risk CKD should be developed and performed regularly as part of a routine checkup. Patients should also be advised to avoid the unnecessary routine use of NSAIDs to relieve pain, to ensure their blood pressure is controlled with antihypertensive medication, and to adhere to therapeutic lifestyle interventions.

## Data Availability

The raw data supporting the conclusions of this article will be made available by the authors, without undue reservation.
